# Urinary human papillomavirus DNA as an indicator of gynaecological infection in young women in *Schistosoma* and HIV endemic South Africa

**DOI:** 10.3389/fgwh.2024.1436064

**Published:** 2025-01-23

**Authors:** P. Pillay, H. N. Galappaththi-Arachchige, M. Taylor, B. Roald, E. F. Kjetland

**Affiliations:** ^1^ Department of Biomedical and Clinical Technology, Faculty of Health Sciences, Durban University of Technology, KwaZulu-Natal, South Africa; ^2^ Faculty of Medicine, University of Oslo, Oslo, Norway; ^3^ Discipline of Public Health Medicine, University of KwaZulu-Natal, Durban, South Africa; ^4^ Department of Pathology, Oslo University Hospital, Oslo, Norway; ^5^ Norwegian Centre for Imported and Tropical Diseases, Department of Infectious Diseases, Oslo University Hospital, Oslo, Norway

**Keywords:** urine, human papillomavirus, DNA, cervico-vaginal lavage, female genital schistosomiasis

## Abstract

**Background:**

Globally, Africa has the highest HIV, cervical cancer and schistosomiasis prevalence. Female Genital Schistosomiasis (FGS) is hypothesized to be associated with HIV and cervical atypia. Young women aged 15 and above, constituting almost 3 million of the South African population, have limited health care access and are at risk for this triad of diseases. Urinary HPV DNA analysis is a non-invasive sampling method that can assist in evaluating risk among this population. This study compared the analysis of HPV DNA in urine and cervico-vaginal lavage (CVL) samples to cytology Pap smear, *Schistosoma* microscopy and HIV results.

**Methods:**

In this cross-sectional study, 235 young women aged 16 years and older from rural high schools in KwaZulu-Natal participated. HPV DNA analysis was done in urine and CVL samples. Pap smears were analysed for squamous cell atypia and urine microscopy was used for the identification of *Schistosoma* ova.

**Results:**

Urinary schistosomiasis was reported in 49 (20.9%) and HIV detected in 49 (20.4%). Urinary and CVL HPV DNA was found in 147 (62.6%) and 177 (75.3%) respectively. Any atypia was detected cytologically among 173 (73.6%). The following associations were found using the Pearson Chi-Square and a Likelihood Ratio test: (a) between HIV positive status and urinary HPV DNA positive cases on both the urine (*X*^2^ = 5.007; *p*-value = 0.025) and (*X*^2^ = 4.264; *p*-value = 0.039) and between HIV positive status and CVL HPV DNA tests respectively (*X*^2^ = 5.165; *p*-value = 0.023) and (*X*^2^ = 4.321; *p*-value = 0.015), and (b) among urine HPV DNA and the CVL HPV DNA tests, where (*X*^2^ = 52.966; *p*-value = 0.001) and (*X*^2^ = 50.716; *p*-value = 0.001). Urine HPV DNA showed a sensitivity of 75.7% and specificity of 77.6% relative to the CVL HPV DNA. There was no statistical association between urinary schistosomiasis and HPV or with any atypia.

**Conclusion:**

Urine has the potential of being optimized as an alternative and possibly more acceptable sample for HPV detection among young adolescent populations at risk in comparison to CVL samples. An integrated targeted intervention incorporating *Schistosoma* in addition to HPV and HIV testing needs consideration among young women in this age group from endemic areas.

## 1 Background

Globally, Africa has the highest prevalence of HIV, cervical cancer and schistosomiasis (1–3). Female genital schistosomiasis (FGS) is hypothesized to be associated with both HIV and HPV (4–6). Human papillomavirus (HPV) is a well-established causal agent for anogential cancers (vulva, vagina, cervix, and anus) among women, with most invasive cancers being associated with high risk (HR) HPV types 16 and 18 (7). It has been reported that women aged 15 and older who are at greatest risk for acquiring HPV make up 21.9 million of the South African population (8). Risks for HPV and other sexually transmitted infections (STIs) increase at the age of sexual debut. Unsafe sex practice was reported to be the leading risk factor requiring further public health intervention (9). It is also established that HIV positive women have an increased risk for cervical cancer (10). While HPV is known to regress in some women, the rate of regression among those who are also at risk for HIV pose additional challenges (11). Strategies to reduce cervical cancer include screening programmes, however in low to middle income countries like South Africa where the burden is high coupled with HIV, the cervical screening policy, has only recently been amended to accommodate HIV positive women (12). Another strategy that has been adopted is the HPV vaccination programme which has been rolled out in primary schools across South Africa in 2014, targeting girls aged 9–12 years, however many young women older than the vaccination age (typically the participants included in this study), but lower than the cervical screening age particularly with the same profile as the study participants remain at risk.

In *Schistosoma* endemic areas, it has been suggested that schistosomiasis could be a risk factor for acquisition and maintenance of HPV, thus being a co-factor for the development of cervical cancer, a major health burden in developing countries (5). The highest disease burden of HIV, HPV and FGS is borne by adolescent girls and women in sub-Saharan Africa (SSA), who are affected in their reproductive years (13). Mortality of women from HIV and cervical cancer in turn has detrimental impacts on the lives of their children and families (14).

In schistosomiasis endemic areas, urine samples are collected for microscopy. These samples commonly also contain cervical epithelial cells (which may be HPV DNA infected) as “contaminants” from the genital tract due to the close proximity of the urethra to the genital tract (15). It is in these cells originating from the genital tract that the HPV DNA can be detected in urine samples. In light of the challenges with conventional cervical cancer screening and the ethical implications of attaining genital samples from young women, HPV DNA analysis of urine samples may be a more socially acceptable, non-invasive diagnostic tool for monitoring and evaluating the HPV risk as well as to monitor the follow up of young women post HPV vaccination (16). The aim of the present study was to investigate HPV DNA analysis using polymerase chain reaction (PCR) in more easily obtainable urine samples from young women aged 16 years and above, to assess the risk of cervical cancer in schistosomiasis endemic areas.

## 2 Methods

### 2.1 Study population

This research project is nested in a large clinical study of FGS on young women, aged 16 years old and above, attending high schools in the Ugu, King Cetshwayo and Ilembe Districts, KwaZulu-Natal. Within the province of KwaZulu-Natal, Ugu District is situated along the coast south of Durban and has a population of 754.000 people, 38% are below the age of 14 years and 52% are female (17). King Cetshwayo and Ilembe Districts are situated on the north coast of KwaZulu-Natal. The King Cetshwayo District hosts a population of approximately 982,726 people. Females make up 52.6% of the population and 49.8% of the households are headed by women (17). The Ilembe District has a population of 678,048 people and comprises 52% of women (17). The areas included are endemic for both schistosomiasis and HIV. A subsample of 235 young women aged 16–23 years from the larger sample was selected for HPV DNA analysis in urine and cervico-vaginal lavage. This convenience sample of young women was selected because they were part of a cohort that were followed up.

### 2.2 Sample collection and storage

The urine and genital samples were collected from young sexually active women. Consenting participants underwent a semi-structured face-to-face interview in the local language isiZulu and urine samples for microscopy were collected and processed locally, prior to gynaecological examination. Cervico-vaginal lavages were collected by spraying 10 ml saline on the vaginal wall and cervix twice, and then drawn back into the syringe. Thereafter, 1 ml of each sample was then dispensed into labelled cryotubes. Pap smears were collected by scraping a wooden spatula in the cervix and the fornices, the material then smeared onto a slide which was then spray-fixed using a commercial cytological fixative for preservation and further analysis using cytology. The detailed procedures for data and sample collection and other analyses, for urine and genital samples have been described previously (18–20). HPV DNA analysis was conducted on collected urine and CVL samples. The collected samples for DNA analysis in this study were stored at −80 °C as per the storage protocols for future DNA analysis (21).

### 2.3 Laboratory analyses

#### 2.3.1 HPV-DNA

Samples were prepared using the Abbot m2000 RealTime (Abbot) system for DNA extraction, and the Real Time High-Risk HPV test for detection. This assay is able to detect and type 16 and 18 as high-risk DNA types, in addition the following genotypes were detectable as high-risk HPV but not typed: 31, 33, 35, 39, 45, 51, 52, 56, 58, 59, 66 and 68. For both urine and cervico-vaginal lavage, 500 µl of sample was processed using the Abbot mSample Preparation_DNA_ System. Once DNA was isolated, samples were then ready for the amplification master mix and thereafter the Abbott RealTime HR-HPV assay protocol was followed (22). In the present study, the PCR analysis was conducted at a private accredited diagnostic laboratory in South Africa using their calibrated routine protocol for HPV DNA analysis.

#### 2.3.2 Urine microscopy

Urine samples were processed in the field site laboratory by a laboratory technician. Urine samples for microscopy were preserved with 1 ml of 2% tincture of merthiolate in 5% formalin solution (23). The samples were spun for 10 min at 4,000 rates per min (rpm) and the sediment examined microscopically, magnification with objective 10, by trained field workers. The samples underwent quality control by an independent microscopist on 10% of randomly chosen samples.

#### 2.3.3 Cytology

The Pap smears were stained and evaluated by a trained cytologist and reported in accordance with the Bethesda system (24). This system uses the following categories for atypia which could be reported as either atypical squamous cells of undetermined significance (ASCUS), low grade squamous intraepithelial lesion (LSIL) and high grade squamous intraepithelial lesion (HSIL). Each of these categories includes HPV. ASCUS is an entity which is used when the distinction between reactive changes or LSIL is difficult to determine cytologically (25). For the analysis in the present study, the category of “any atypia” was used to describe the collective sum of the ASCUS, LSIL and HSIL cases.

### 2.4 Data analysis

The aim of this study was to investigate whether the urinary HPV DNA could be an indicator of gynaecological infection in young rural women in *Schistosoma* and HIV endemic South Africa. As such, a questionnaire was used as the primary source of data collection in this study as well as the results from laboratory analysis. Laboratory analysis included the urine and CVL HPV DNA testing, urine microscopy, cytology and other STI results. Following the data collection, the researchers cleaned, organized, and then analysed the data using SPSS software (version 28.0). To analyse the collated data and demonstrate the findings of this study, the researchers used both descriptive and inferential statistical matrices.

Descriptive statistical techniques were used to measure the central tendencies and dispersion among variables. On the other hand, to demonstrate the relationship among variables and assess the degree of association, probability, and nature of relationship among variables in the dataset, inferential statistical techniques were used. All calculated inferential tests were based on the traditional significance level of *p* = <0.05.

## 3 Results

### 3.1 Respondents' profile and schistosomiasis risk factors

**Figure 1:**
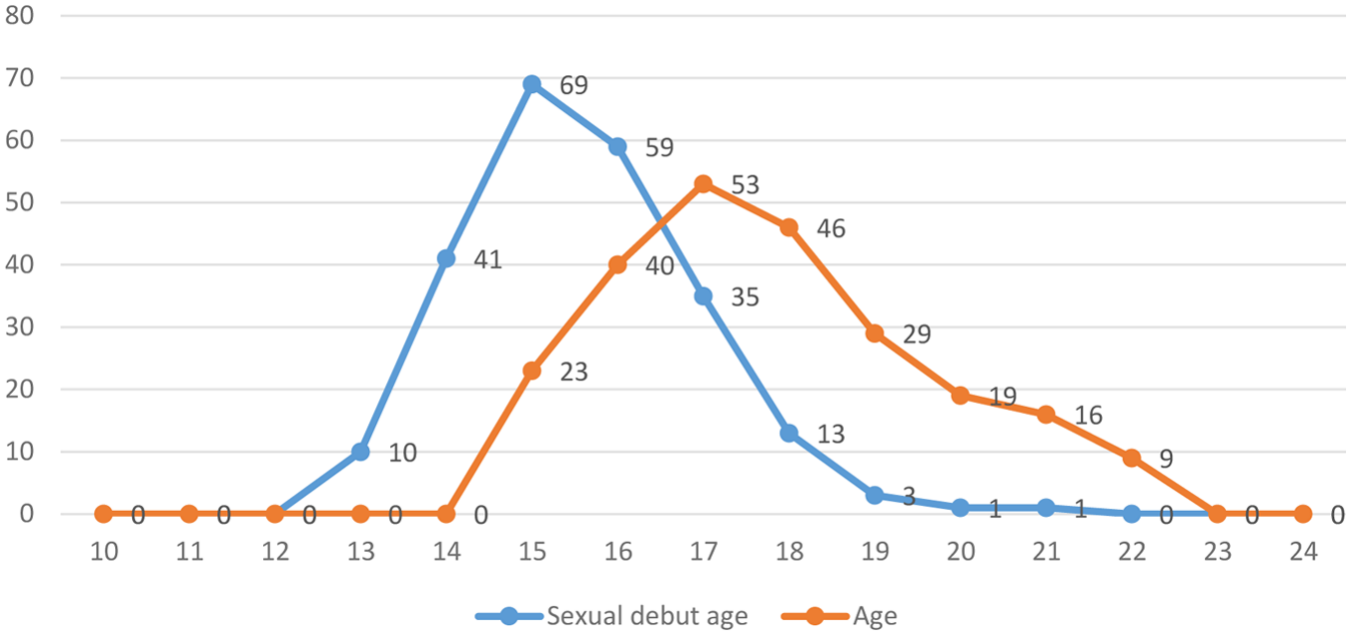
Shows respondents’ sexual age debut and respondents’ age.

The results in Figure 1 and Table 1 show that most of respondents are either 18 or more years old (*n* = 173; 73.2%), with a mean (M) age of 18.78 years and a standard deviation (SD) of 1.85 years. Likewise, the results in Table 1 and Figure 1 show that most of the young women began their sexual debut before the age of 18 (*n* = 179; 76.2%), where the M = 16.34 years and SD = 2.30 years. The results in Figure 1 further indicates that the minimum and maximum sexual age debut was 14 years and 22 years, respectively. The majority of the study participants indicated that they had only one sexual partner during the last month (*n* = 143; 60.9%).

**Table 1:** Shows demographic profile and the exposure to risk factors for schistosomiasis and sexually transmitted infections (*n* = 235).

Variables	Frequency (*n*)	Percent (%)
Age		
≤17	63	26.8
≥18	172	73.2
Sexual debut age		
≤17	179	76.2
≥18	53	22.6
Undisclosed	3	1.3
Number of sexual partners in the last month		
None	89	37.9
One partner	143	60.9
Undisclosed	3	1.3
Number of children alive		
Non-alive	13	5.5
One	106	45.1
Two	10	4.3
Never had a child	106	45.1
HIV status		
Negative	187	79.6
Positive	48	20.4
Type of water body exposed to		
Tap-water	8	3.4
River	208	88.5
Dam	13	5.5
Standing water	1	0.4
Several	5	2.1
Bathe/wash in River/Dam water		
Never	33	14.0
Rarely	13	5.5
Sometimes	81	34.5
Often	61	26.0
Daily	47	20.0
Wash blankets in Riverine/Dam water		
Never	32	13.6
Rarely	26	11.1
Sometimes	89	37.9
Often	77	32.8
Daily	11	4.7
Plays/swims in Riverine/Dam water		
Never	68	28.9
Rarely	21	8.9
Sometimes	82	34.9
Often	46	19.6
Daily	18	7.7
Does laundry in Riverine/Dam water		
Never	25	10.6
Rarely	6	2.6
Sometimes	51	21.7
Often	104	44.3
Daily	49	20.9

Since there are several water sources that serves as risk factors for schistosomiasis among rural women, the results in this study revealed that most of the respondents (*n* = 208; 88.5%) had come in contact with water from the river and about (*n* = 13; 5.5%) indicated that they had come in contact with water from dams. The results for the frequencies of their contact and activities (such as laundry, bathing, swimming or playing) with these water bodies are detailed in Table 1.

### 3.2 Detection of HPV DNA, cytology and urinary schistosomiasis

The Real Time High-Risk HPV test was able to identify and type HPV-16 and HPV-18 as well as the following range of other high-risk DNA genotypes comprising the following: HPV-31, 33, 35, 39, 45, 51, 52, 56, 58, 59, 66 and 68. All the respondents in this study were tested for HPV DNA using CVL and urine, for any atypia using cytology Pap smears and for schistosomiasis using microscopy as shown in Table 2. The results from the PCR analysis revealed that (*n* = 177; 75.3%) of the participants tested HPV DNA positive in CVL, and (*n* = 147; 62.6%) of the sample tested positive HPV DNA in urine, while (*n* = 173; 73.6%) were tested positive for any atypia using cytology samples.

**Table 2:** Shows the results for HPV DNA, atypia and schistosomiasis (*n* = 235) .

Variables	Frequency (*n*)	Percent (%)
Human papillomavirus (HPV) DNA and Any atypia		
Cervico-lavage (HPV DNA)		
Negative	58	24.7
Positive	177	75.3
Urine (HPV DNA)		
Negative	88	37.4
Positive	147	62.6
Cytology any atypia		
Negative	62	26.4
Positive	173	73.6
Schistosomiasis		
Urine microscopy		
Negative	186	79.1
Positive	49	20.9

Of the 75.3% HPV DNA cervico-vaginal lavage positive participants, 51.5% of them were positive for other high-risk HPV, 12.5% were positive for HPV 16 and other high-risk-HPV, 4.7% were positive for HPV 18 and other high-risk HPV, 3.0% were positive for HPV 16, 2.1% were positive for HPV 18, and 1.3% were positive for HPV 16, HPV 18 and other high-risk HPV types. Of the 62.6% of the urine HPV PCR positive participants, 43.0% of them were positive for other high-risk HPV, 9.8% tested positive for HPV 16 and other high-risk HPV, 4.3% were positive for HPV 18 and other high-risk HPV, 3.0% were positive for HPV 18, 2.1% were positive for HPV 16, and 0.4% were positive for HPV 16, HPV 18 and other high-risk HPV.

Furthermore, all the respondents in this study were tested for schistosomiasis using urine microscopy as shown in Table 2. Here, the urine microscopy diagnostic results revealed that (*n* = 49; 20.9%) of the participants tested positive for schistosomiasis.

### 3.3 Urinary HPV DNA detection in comparison with the respondents' profile, sexual behaviour and sexual health

The results of the urine PCR test identified 147 cases (62.6%) as positive for HPV (Table 2). Of the 147 urine HPV DNA positive cases, 48 (76.2%) were ≤17 years old, 117 (65.4%) had their sexual debut at ≤17 years old, 34 (77.3%) were tested HIV positive, 60 (67.4%) had no sexual partner at the time of data collection, and 85 (59.4%) of them had one sexual partner at the time of the data collection (see Table 3).

**Table 3:** Shows the cytology and polymerase chain reaction (PCR) test results for HPV and any atypia relative to respondents’ profile, sexual behaviour and sexual health (*n* = 235).

Factors	Urine HPV PCR		95% CI	Any atypia cytology		95% CI	Cervico-vaginal lavage HPV PCR		95% CI
	Negative	Positive		Negative	Positive		Negative	Positive	
Age			−0.326 to 0.066			−0.012 to 0.269			−0.171 to 0.113
≤17	15 (23.8%)	48 (76.2%)		21 (33.3%)	42 (66.7%)		15 (23.8%)	48 (76.2%)	
≥18	73 (42.4%)	99 (57.6%)		41 (23.8%)	131 (76.2%)		43 (25.0%)	129 (75.0%)	
Sexual debut age			−0.264 to 0.009			−0.082 to 0.155			−0.158 to 0.112
≤17	62 (34.6%)	117 (65.4%)		48 (26.8%)	131 (73.2%)		43 (24.0%)	136 (76.0%)	
≥18	25 (47.2%)	28 (52.8%)		14 (26.4%)	39 (73.6%)		14 (26.4%)	39 (73.6%)	
Still a virgin	1 (33.3%)	2 (66.7%)		0 (0.0%)	3 (100.0%)		1 (33.3%)	2 (66.7%)	
Sexual partners			−0.188 to 0.092			0.010–0.296			−0.030 to 0.146
None	29 (32.6%)	60 (67.4%)		32 (36.0%)	57 (64.0%)		24 (27.0%)	65 (73.0%)	
One partner	58 (40.6%)	85 (59.4%)		30 (21.0%)	113 (79.0%)		33 (23.1%)	110 (76.9%)	
Still a virgin	1 (33.3%)	2 (66.7%)		0 (0.0%)	3 (100.0%)		1 (33.3%)	2 (66.7%)	
Children alive			−0.055 to 0.229			−0.298 to −0.010			−0.194 to 0.086
Non-alive	2 (15.4%)	11 (84.6%)		5 (38.5%)	8 (61.5%)		1 (7.7%)	12 (92.3%)	
One	46 (43.4%)	60 (56.6%)		21 (19.8%)	85 (80.2%)		25 (23.6%)	81 (76.4%)	
Two	5 (50.0%)	5 (50.0%)		3 (30.0%)	7 (70.0%)		2 (20.0%)	8 (80.0%)	
Never had a child	35 (33.0%)	71 (67.0%)		33 (31.1%)	73 (68.9%)		30 (28.3%)	76 (71.7%)	
HIV status			0.029–0.270			0.012–0.245			0.019–0.260
Negative	78 (40.8%)	113 (59.2%)		55 (28.8%)	136 (71.2%)		53 (27.7%)	138 (72.3%)	
Positive	10 (22.7%)	34 (77.3%)		7 (15.9%)	37 (84.1%)		5 (11.4%)	39 (88.6%)	

In addition, the cytology results for any atypia identified 173 cases (73.6%) as positive for either ASCUS, LSIL or HSIL. These positive results were obtained in cytology samples from 173 suspected HPV participants. Of these 173 positive cases, 131 (76.2%) were ≥18 years old, 131 (73.2%) had their sexual debut at ≤17 years old, 37 (84.1%) were tested positive for HIV, 113 (79.0%) of them had one sexual partner at the time of data collection, and 57 (64.0%) had no sexual partner when the data was collected.

On the other hand, the results of the PCR examination using the CVL identified 177 (75.3%) as positive cases for HPV from the sample (Table 2). Of the 177 positive cases, 129 (75.0%) were ≥18 years old, 136 (76.0%) had their sexual debut at ≤17 years old, 39 (88.6%) were tested positive for HIV, 110 (76.9%) had one sexual partner at the time of data collection, and 65 (73.0%) had no sexual partner at the time of data collection (see Table 3).

These results further suggested that there is a significant statistical relationship between HPV and the respondents’ profile, sexual behaviour and sexual health. As determined by a Pearson Chi-Square test and a Likelihood Ratio test, the results suggested that there is a strong link between respondents' age category and urine HPV DNA, with (*X*^2^ = 6.834; *p*-value = 0.009) and (*X*^2^ = 6.062; *p*-value = 0.014), respectively. Thus, these findings support the descriptive results of this study, and suggest that participants of lower ages (≤17 years old) are associated with positive cases of HPV based on urine PCR. This is an indication that younger respondents are more likely to be tested positive for HPV DNA in urine. Similarly, an association was found between respondents' number of sexual partners and HPV. The results suggested that there is a strong statistical significance between respondents with one active sexual partner and positive cases of any atypia cytology and urine HPV DNA diagnostics, where (*X*^2^ = 7.424; *p*-value = 0.024) and (*X*^2^ = 8.027; *p*-value = 0.018) as determined by a Pearson Chi-Square test and a Likelihood Ratio test, respectively. As such, these findings support the descriptive results of this study, and suggest that participants with active sexual partners are associated with positive cases of HPV DNA and any atypia as diagnosed by cytology. In a similar fashion, the results revealed that there is a statistical association between respondents' HIV positive status and HPV positive cases based on both the urine and the CVL PCR tests. The results from the Pearson Chi-Square test and the Likelihood Ratio test suggested that there is a strong link between respondents' HIV positive status and HPV DNA diagnostics using urine, with (*X*^2^ = 5.007; *p*-value = 0.025) and (*X*^2^ = 4.264; *p*-value = 0.039), respectively. Similarly, the findings from the Pearson Chi-Square test and the Likelihood Ratio test suggested that there is a statistical relationship between respondents' HIV positive status and PCR HPV diagnostics based on the CVL DNA diagnosis, with (*X*^2^ = 5.165; *p*-value = 0.023) and (*X*^2^ = 4.321; *p*-value = 0.015), respectively. Thus, these findings support the descriptive results of this study, and propose that participants with positive HIV status are associated with positive cases of HPV.

No statistical association was found between respondents' age, sexual age debut, number of sexual partners, number of children alive, and HPV positive cases based on the CVL DNA diagnosis. Likewise, no association was found between respondents' sexual age debut, number of children alive and HPV based on urine PCR and cytology.

#### 3.3.1 Sensitivity and specificity of urine HPV PCR in comparison with cervico-vaginal lavage HPV PCR

The findings in Table 4 shows the sensitivity and specificity of urine HPV DNA test of the sample in relation to the HPV DNA diagnoses based on the CVL. The results of the examination using the urine, showed a sensitivity of 75.7% and specificity of 77.6% relative to the CVL HPV DNA test.

**Table 4:** Sensitivity and specificity of HPV urine PCR test in comparison with the HPV cervico-vaginal lavage PCR test (*n* = 235).

Urine HPV DNA	Cervico-vaginal lavage HPV DNA		Total
	Negative	Positive	
Negative Count	45	43	88
% within Urine HPV DNA	51.1%	48.9%	100.0%
% within Cervico-vaginal Lavage HPV DNA	77.6%	24.3%	37.4%
Positive Count	13	134	147
% within Urine HPV DNA	8.8%	91.2%	100.0%
% within Cervico-vaginal Lavage HPV DNA	22.4%	75.7%	62.6%
Total Count	58	177	235
% within Urine PCR	24.7%	75.3%	100.0%
% within Cervico-vaginal Lavage PCR	100.0%	100.0%	100.0%

Using the CVL HPV DNA test as a gold standard, Table 4 also shows the positive predictive value (i.e., the probability that a positive test means that they have the disease) and the negative predictive value (i.e., the probability that a negative test means that they really do not have the disease). These results revealed that using the CVL HPV DNA test, the positive predictive value of the participants that really have the disease was 91.2%; while the negative predictive value of the participants that do not really have the disease was 51.1%.

The results in Table 4 further shows the prevalence of HPV (i.e., the proportion of the participants in the population that have the disease) in the study population was 75.3%. Therefore, the CVL HPV DNA test had a very good measure of agreement (*k* = 0.127; *p*-value = 0.050) relative to the urine HPV DNA test.

Table 5 shows the findings for the sensitivity and specificity of HPV based on any atypia as detected by cytology in relation to the CVL HPV DNA. The results from the any atypia showed a sensitivity of 76.8% and specificity of 36.2% relative to the HPV DNA test using CVL. Using the CVL HPV DNA test as a gold standard, Table 5 equally demonstrates the positive predictive value (i.e., the probability that a positive test means that they have the disease) and the negative predictive value (i.e., the probability that a negative test means that they really do not have the disease). The findings revealed that by using the CVL HPV DNA test, the positive predictive value of the participants that really have the disease was 78.6%; while the negative predictive value of the participants that do not really have the disease was 33.9%. The results in Table 5 further indicates the prevalence of HPV based on any atypia cytology test (i.e., the proportion of the participants in the population that have the disease). These results suggested that the prevalence of HPV in the population was 75.3%. Therefore, the CVL PCR test had a very good measure of agreement (*k* = 0.127; *p*-value = 0.050) relative to any atypia as seen in cytology.

**Table 5:** Sensitivity and specificity of HPV based on any atypia cytology test in comparison with the HPV DNA test (*n* = 235).

Cytology any atypia	Cervico-vaginal lavage HPV DNA		Total
	Negative	Positive	
Negative Count	21	41	62
% within Cytology Any Atypia	33.9%	66.1%	100.0%
% within Cervico-vaginal Lavage HPV DNA	36.2%	23.2%	26.4%
Positive Count	37	136	173
% within Cytology Any Atypia	21.4%	78.6%	100.0%
% within Cervico-vaginal Lavage HPV DNA	63.8%	76.8%	73.6%
Total count	58	177	235
% within Cytology Any Atypia	24.7%	75.3%	100.0%
% within Cervico-vaginal Lavage HPV DNA	100.0%	100.0%	100.0%

In addition, the findings of this study further reveal that there is a significant statistical relationship between the two HPV DNA diagnostic test results. As determined by a Pearson Chi-Square test and a Likelihood Ratio test, the results suggested that there is a strong association between urine HPV DNA and the CVL HPV DNA, where (*X*^2^ = 52.966; *p*-value = 0.001) and (*X*^2^ = 50.716; *p*-value = 0.001), respectively. This is an indication that urine HPV DNA test can be used in placed of the CVL PCR test for diagnosing HPV among women. Similarly, an association was found between any atypia as seen in cytology and the CVL DNA test results, where (*X*^2^ = 3.826; *p*-value = 0.050) as determined by a Pearson Chi-Square test. As such, these findings indicate that any atypia diagnosed by cytology can be use in placed of the CVL DNA test for diagnosing HPV among women. On the contrary, no statistical association was found between urine HPV PCR and urinary Schistosomiasis as detected by microscopy.

## 4 Discussion

Young women in South Africa are known to be at high risk for HIV. To some extent there has been advocacy around HIV over the years, yet the prevalence is still rife. Cervical cancer is also known to be a leading cancer among the South African population, despite it being a preventable disease. In a bid to curb cervical cancer, the South African Department of Health rolled out a school-based HPV vaccination programme among school girls aged 10–12 years in 2014, while this programme has been in existence for several years, there are still several challenges (26). The HPV vaccination programme only targets girls within the specified age, while this has been done in alignment with resources available and other considerations, the HPV disease burden may be continuing amongst those who have missed the vaccination age. The association between HIV and the progression to invasive cervical cancer linked to high risk HPV strains is also another co-factor (11). FGS on the other hand is a highly neglected disease, which is a challenge to diagnose and treat. The link between FGS and HIV has been established as well as the link between HIV and HPV. Convincing evidence that HPV and FGS are associated has however, been more difficult to ascertain thus far.

Based on our findings, the young women included in the study were found to have risk factors for all three of the aforementioned diseases as determined by their demographic profile, history of water contact and sexually transmitted infection status. The risk factors include reported sexual activity among majority of the participants, with the minimum and maximum reported sexual debut age being 14 and 22 years respectively. They also reported varying frequencies and duration of water contact with rivers or dams, which are risk factors for schistosomiasis, with the prevalence of 20.9% detected using microscopy. Based on molecular testing for HPV DNA, the prevalence of HPV infection among our study population was found to be 75.3% and the overall HIV prevalence was 20.4%. Presently routine HIV testing, reproductive health care and deworming programmes are limited or non-existent for young women at risk of these diseases (4).

HPV is known to have a higher prevalence among younger sexually active women in comparison to older women and is known to regress with age in some (27). The extent of regression in a population who are already at risk for HIV, schistosomiasis and other STI's is however, largely unknown (28). Our study revealed that that participants of lower ages (≤17 years old) are associated with positive cases of HPV based on urine PCR—confirming the arguments established in other studies that younger women are more likely to test positive for HPV (in our case HPV DNA in urine) (29). An association was also found in our study between respondents' number of sexual partners and HPV—indicating that there is a strong statistical significance between respondents with one active sexual partner and positive cases of any atypia cytology and urine HPV DNA diagnostics. Consequently, the findings from our study revealed that being HIV positive is strongly associated with HPV DNA diagnostics using urine. Similarly, the results of the current study found that HIV positive status and PCR HPV diagnostics based on CVL DNA diagnosis are strongly correlated. Although, the collection of gynaecological samples in young women, requires logistical infrastructure that is not easily available in the field. The aim of this study therefore was to determine if urine, which is a relatively easy sample to collect can be used as an indirect indicator for HPV DNA detection among young women also at risk for FGS.

### 4.1 Urine HPV PCR analysis

In these young South African women, we found that almost three quarters of the study population tested positive for HPV DNA and based on our results, urine analysis was a good predictor for HPV positivity in gynaecological samples. This has been seen in several prior studies using various types of gynaecological and urine samples either clinician collected or self-collected (30–32). In the present study, correlation of HPV DNA detection among CVL and urine samples indicated that urine could be used as an alternate sample to CVL for HPV detection since there was a good measure of agreement (k = 0.127; *p*-value = 0.050) relative to the urine HPV DNA test.

When investigating the link between risk factors such as age and age of onset of sexual activity and HPV, active sexual partners and HIV, associations were found with urine detected HPV and or squamous atypia. Our investigation of the risk factors however did not take into account the sexual activity of the respondent's partners, who are equally responsible for transmitting HPV, HIV and other STI's as well.

While it is known that HPV can regress, it has been found that HR HPV strains are more likely to persist and progress to invasive cervical cancer and it is also known that women with HIV are more likely to have HR HPV strains (28). In the present study the following HR strains were detected: HPV 16, 18 as well as the following range of “other high-risk” genotypes: 31, 33, 35, 39, 45, 51, 52, 56, 58, 59, 66 and 68, among 75.3% and 62.6% of the women in their CVL and urine samples respectively. These strains have been identified to have varying progression rates to invasive cancer (33). A point of concern is that women in this age range, who have not qualified or missed HPV vaccination, do not qualify for cervical screening and are also at risk for HIV, may be at a higher risk for progression to invasive cancer of the cervix.

The South African cervical cancer screening programme has been in existence for 23 years, which was traditionally limited to women aged 30 and above. It was recently amended to accommodate HIV positive women so that all HIV positive women are able to be screened for cervical cancer at diagnosis and if found to have any positive atypia, they will be screened annually and if negative every three years as per the WHO guidelines (8, 12).

Younger women have limited reproductive healthcare options in South Africa, and many do not know their HIV status to be eligible for cervical screening despite their known proneness to acquiring HIV and other STIs. In addition to cervical cancer progression, young women are prone to have teenage pregnancies and are at risk for adverse pregnancy related events including HIV transmission from mother-to-child (29, 34). While HPV vaccination was rolled out in South Africa in 2014 to primary school girls aged 9–12, its impact on cervical cancer may only be seen in years to come (8, 12).

Consequently, based on the preliminary findings from the present study using urine to detect HPV DNA could be an alternative to genital sampling among women in resource limited settings—since the results of our study show that urine HPV DNA correlated well with CVL HPV DNA (35). Urine is an easily obtainable sample and our findings of 75.7% sensitivity and 77.6% specificity with the additional good measure of agreement with the CVL HPV DNA testing has potential to be optimized for HPV testing using more cost-efficient methods and applied in low resource countries amongst high risk populations. In the present study the HPV DNA analysis was performed on samples post collection in a laboratory setting, however there are newly available point of care diagnostic methods which could be used in clinics (36). In some low resource countries, where conventional screening with cytology is limited, HPV testing has been adopted as part of a screen and treat campaigns. HPV DNA testing has the potential to provide results with a faster turn-around time and has been found to have superior sensitivity to conventional cytology (37). In addition to HPV DNA testing, the WHO recommends onsite treatment and follow-up facilities (35). One of the drawbacks to caution against is over-treatment for otherwise reversible HPV infection along with the risk of HIV progression due to the adverse effects of the treatment among women with HIV (38).

### 4.2 HPV and schistosomiasis

While the natural progression to invasive cancer is known to take years to decades, maintaining a balance in terms of avoiding over-treatment or misdiagnosis of lesions is required. In women who are at risk for FGS it is necessary to ensure that FGS is considered a clinical differential diagnosis to cervical cancer and pre-cancer, since FGS often is misdiagnosed or underdiagnosed (39). Clinical lesions on the cervix due to HPV or cervical cancer and FGS may be difficult to distinguish clinically. While it is hypothesized that there is a possible association of FGS and cervical atypia it has been difficult to prove a strong association between these entities. In the present study there was no distinct association between the two entities. However, due to the genital tract being a common site for these two entities, we cannot ignore either and therefore need to ensure that we are able to differentiate them. Treatment, requires resources and infrastructure (20).

While there was no statistical association in the present study between urinary schistosomiasis and HPV, it must however be noted that genital schistosomiasis has been found to be associated with HIV in several studies, this cannot be ignored due to the geographical overlap in disease burden with these diseases and HIV (5, 40). FGS is also a neglected entity which is rarely diagnosed, this is confounded by limitations in health care workers' knowledge of the entity at public health facilities, limited resources, limited knowledge among women at risk, and the reduced health seeking behaviour amongst women for STIs and other genital symptoms (5, 41, 42). This is possibly why no association between schistosomiasis and squamous cell atypia was found due to the small sample size used in the present study.

Validating the use of urine samples for HPV DNA and *Schistosoma* DNA genotyping using point of care testing could be used as an alternative to genital sampling. Such point of care tests could be useful in providing DNA evidence of the infective agents to inform the diagnosis. Validation with rapid point of care HPV and *Schistosoma* DNA testing will be useful since such testing could be used to complement other diagnostic tests such as colposcopy and imaging techniques that are being developed for FGS (43).

### 4.3 HPV and HIV

Considering the supporting finding that there was a statistical association between respondents’ HIV positive status and HPV PCR positive cases based on both the urine and the cervico-vaginal lavage, warrants intervention. It may be argued that HPV in its natural course has the tendency to clear, but it has been found that this tendency is halved among people co-infected with HIV, likewise clearance is decreased in women with FGS (44). In turn women infected with HIV and HPV have been found to progress much quicker to invasive cancer (44). In young HIV positive women, who may not be able to get access to gynaecological examinations, urine HPV analysis may be a practical way of identifying those with HR HPV strains in order to refer them for further management. Cervical cancer is treatable when detected in the pre-invasive stages, however, sadly, for most women in low to middle income countries, it is only detected when it is invasive and the survival chances are limited. Furthermore, with the advent of HIV and other co-factors, cervical cancer is now ranked third most common in women 45 years and younger (45).

### 4.4 Study limitations

The urine and cervico-vaginal lavage samples were collected several years ago and were in storage at −80 °C, it is possible that the samples used in this investigative study were not optimal, however it is well documented that DNA, can be preserved for several years (21) The sample was also a convenience sample, larger studies are recommended to fully validate urine as an alternative to gynaecological samples. The laboratory testing was done at a private diagnostic laboratory, and the current assay was validated for gynaecological samples and not validated for urine PCR. However, urinary HPV DNA analyses showed an association with the cervico-vaginal lavage results and there was a good measure of agreement between the tests. The cytology results were not validated with histology. Due to this being a cross- sectional study, it was not possible to determine in those who were both positive for HIV and HPV, which disease had occurred first.

## 5 Conclusion

While it may be possible to raise awareness on HIV transmission, and to offer counselling and antiretroviral therapy to try to reduce HIV transmission, coupled with mass anti-schistosomal treatment to these young women, they are currently neglected when it comes to HPV or cervical cancer awareness and treatment options. This is simply because the cohort of young women like those included in this study, do not meet the criteria for HPV vaccination as per South African policy.

Using urine samples as an alternative to genital sampling may be promising, to assess the risk of HPV, though DNA analysis. It is recommended that young women especially in the 16–24 age group who are from *Schistosoma* endemic areas are targeted with specific public health interventions to increase their awareness of this triad of diseases for which they are at risk for. Efforts to raise awareness of HPV and schistosomiasis at community and school level are vital. Strategies to strengthen health seeking behaviour and reproductive health services specifically geared for adolescent women aged 16 and above should be committed to by the Department of Health (to upscale youth friendly reproductive health services) and the Department of Education (to upscale reproductive health literacy at high school level) respectively. Additionally, further research needs to be done to develop point of care urine HPV and *Schistosoma* assays, which may aid in rapid diagnosis. It will also be necessary for the anti-parasitic treatment to be provided in schools and HPV vaccination to be available to not only girls aged 9–12, but to boys as well and to consider vaccination among the slightly older age group of women who are also at high risk.

While adolescent health services do exist in South Africa, they are limited in number and also not optimally utilised by youth (46). While HIV services and family planning services may exist in adolescent health facilities, services related to cervical cancer and FGS are limited.

## Data Availability

The raw data supporting the conclusions of this article will be made available by the authors, without undue reservation.
